# New Theoretical Model of Nerve Conduction in Unmyelinated Nerves

**DOI:** 10.3389/fphys.2017.00798

**Published:** 2017-10-12

**Authors:** Tetsuya Akaishi

**Affiliations:** ^1^Department of Neurology, Tohoku University Graduate School of Medicine, Sendai, Japan; ^2^Department of Neurology, National Hospital Organization Yonezawa Hospital, Yonezawa, Japan

**Keywords:** unmyelinated fibers, conduction velocity, theoretical neuroscience, sodium channel density, cations, anions, electrostatic interactions

## Abstract

Nerve conduction in unmyelinated fibers has long been described based on the equivalent circuit model and cable theory. However, without the change in ionic concentration gradient across the membrane, there would be no generation or propagation of the action potential. Based on this concept, we employ a new conductive model focusing on the distribution of voltage-gated sodium ion channels and Coulomb force between electrolytes. Based on this new model, the propagation of the nerve conduction was suggested to take place far before the generation of action potential at each channel. We theoretically showed that propagation of action potential, which is enabled by the increasing Coulomb force produced by inflowing sodium ions, from one sodium ion channel to the next sodium channel would be inversely proportionate to the density of sodium channels on the axon membrane. Because the longitudinal number of sodium ion channel would be proportionate to the square root of channel density, the conduction velocity of unmyelinated nerves is theoretically shown to be proportionate to the square root of channel density. Also, from a viewpoint of equilibrium state of channel importation and degeneration, channel density was suggested to be proportionate to axonal diameter. Based on these simple basis, conduction velocity in unmyelinated nerves was theoretically shown to be proportionate to the square root of axonal diameter. This new model would also enable us to acquire more accurate and understandable vision on the phenomena in unmyelinated nerves in addition to the conventional electric circuit model and cable theory.

## Introduction

**Figure 1:**
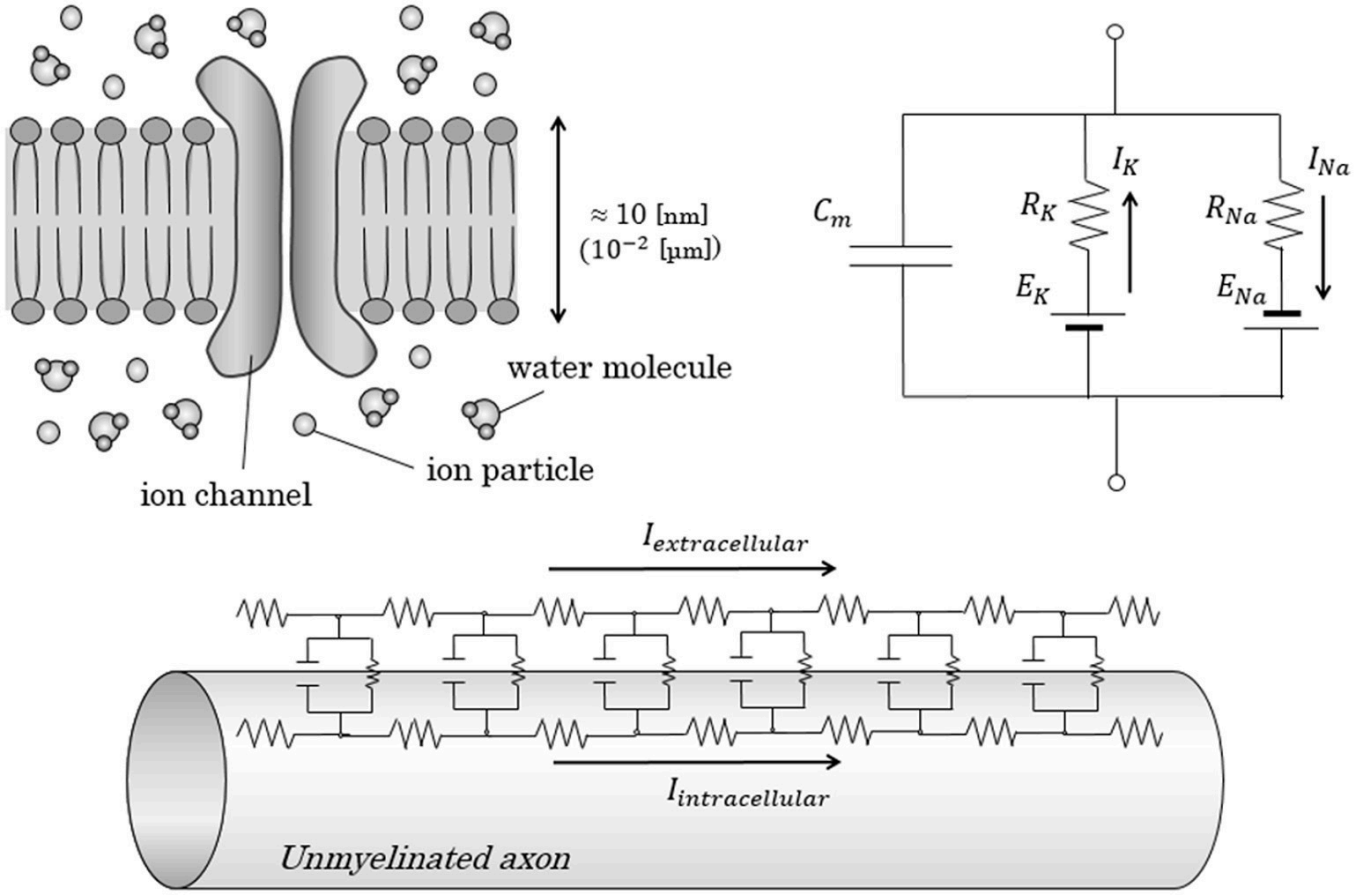
Conventional equivalent circuit model in axonal conduction. Figures show the conventional equivalent circuit model, proposed by Hodgkin and Huxley. (**Upper**) The lipid bilayer itself was regarded as an insulator; the electrolytes inside and outside the lipid bilayer were regarded as a capacitor with a capacitance of *C*_*m*_. Transfer of electric charges penetrating the membrane is expressed as an electric current with ion-specific resistance and ion-specific potential. (**Lower**) In the cable theory, parallel alignment of above-described circuit is proposed to explain the longitudinal conduction in unmyelinated nerves. *C*_*m*_, Capacitance of axon membrane; E, electromotive force; I, intensity of electric current; R, resistance.

The generation and conduction of an action potential on the surface of an axonal membrane has been described as a physiological electric circuit, known as an “equivalent circuit model” (Figure 1; Hartline and Colman, 2007). This fundamental circuit model was first introduced by Hodgkin and Huxley; in this model, movement of an electric charge penetrating through the membrane was described as a parallel alignment of conductance for each type of ion (Hodgkin and Huxley, 1952).

On the basis of this equivalent circuit model, the conduction of the nerve is often described by using a theory in which the conduction is regarded as an electric current on imaginary electric longitudinal leads running inside and outside the axon membrane, known as a cable theory (Koch, 1984). In this theory, based on Ohm's law and Kirchhoff's law, the membrane potential at a specific position and time (*V*_*M*_(*x, t*)) can be described by the equation shown below, known as a cable equation.

(1) $$\begin{array}{r} \lambda^{2}\cdot \frac{\partial^{2}V_{m}\left(x,t\right)}{{\partial x}^{2}}-\tau_{m}\cdot \frac{\partial V_{m}\left(x,t\right)}{\partial t}-V_{m}\left(x,t\right)=0 \end{array}$$

*(*λ*: space constant*, τ_*m*_*: time constant)*

With this model, we can simulate the spatial, time-dependent expansion of the membrane potential on an axon membrane. These conventional, established models enable us to explain and reproduce the actual electric phenomena taking place in axons. However, we have to solve the above-mentioned partial differential equation of second order in the conventional models, and we usually need to apply computer simulations to solve it.

The generation of action potential in an unmyelinated axon is now understood to be realized by ionic transfers through many types of ion channels and resulting ionic concentration gradients across an axon membrane (Gadsby, 2009). However, these concepts had not been fully elucidated in the era of Hodgkin and Huxley; thus, characteristics of ion channels had not been fully incorporated in the conventional models. With the present knowledge of physiology, membrane charges are known to be produced as a result of ionic concentration gradient around ion channels on an axon membrane (Gadsby, 2009). Also, we now know that the carriers of electric charges in the actual site on an axon membrane are the electrically-charged ions. In an electric circuit connected using a metal lead, the electric carrier would be free electrons inside the metal. On the other hand, in axons, free electrons within an electrolyte solution are much fewer than ion particles. Also, the resistance of the lipid bilayer, which is known to be practically an insulator, is too high (500–10,000 [Ω/cm^2^]) to freely carry electric charges within it compared to intra- and extra-cellular fluids (Bennett et al., 1959; Turner and Schwartzkroin, 1980; Shepherd et al., 1985).

Given these recent insights, new models of nerve conduction, in addition to the conventional equivalent circuit model and cable theory, would be desirable.

In this report, from the viewpoint of molecular movements within axons at the nano-scale to pico-scale orders, we will attempt to establish a new type of nerve conduction model for unmyelinated nerves. We will estimate the validity of this new model theoretically and mathematically and see how well it can explain the characteristics of unmyelinated nerves observed in previous studies.

## Materials and methods

### Model of action potential conduction on the surface of an axonal membrane

An action potential is mainly produced in neurons by the influx of sodium ions from the extracellular fluid to the intracellular fluid through voltage-gated sodium (NaV) channels (Lai and Jan, 2006; Hu et al., 2009). Differing from a closed electric circuit with a metal lead, in which the bearer of the electric charge is free electrons, the bearer of the electric charge in the intracellular fluid is multiple electrolyte ions (e.g., sodium, potassium, or chloride ions).

The resistivity of the axonal membrane (lipid bilayers) is far larger than that of the intracellular fluid; thus, the conduction of some kinds of electric charges within lipid bilayers is unrealistic (Naumowicz et al., 2003). It is known that the membrane interior between lipid bilayers is positively charged, because of the molecular polarity of phospholipids (Berkovich et al., 2012). However, this space, less than 1 [nm] width, is too narrow to allow the electrolytes (ionic radius around 100–200 [pm]) to travel freely (Gurtovenko and Anwar, 2009). In the first place, hydrophobic molecules are facing close to each other in this space; thus, electrolytes are unlikely to exist at sufficient level within this space. Considering these facts together, in this report, lipid bilayers are regarded as insulators and are unable to transfer electric currency within them.

Another concern is that whether the inflowing sodium ion particles could be directly transmitted as carriers of electric charges from one NaV channel to the next or not. The ionic migration velocity within a solution is known to be very slow, usually far less than 1.0 [mm/s]. As described in the next section, the allowed time lapse at each NaV channel in nerve conduction is much less than 10^−6^ [s] (= 1.0 [μs]). The velocity of ionic migration could be accelerated to some extent by Coulomb force from the inflowing sodium ions, but it would not be enough to make the ion particles to travel all the way to the next NaV channel within such a short time period. Thus, the transmission of membrane potential from one NaV channel to the next would be realized not by the migration of the inflowing sodium ions all the way to the next channel, but by the transmission of some remote forces like Coulomb force along the surface of an axon membrane.

### Allowed time lapse in one NaV channel

As described in a later section, the density of NaV channels on the axon membrane of unmyelinated nerves is known to be 5–50 [channels/μm^2^](Hu and Jonas, 2014); suggesting that the distance between each of the adjacent NaV channels is less than 0.50 [μm]. Now, we will consider an unmyelinated nerve with an axonal length of 10 [cm] (= 10^5^ [μm]). The estimated number of NaV channels on a longitudinal straight line from the neuron body to a synapse is at least 2.0 × 10^5^ [channels / longitudinal line]. Here, the conduction velocity of unmyelinated nerves of an average size of diameter is known to be around 1.0 [m/s] (= 10^6^ [μm/s]); which means only 0.1 [s] (= 10^2^ [ms]) would be required to travel across an axon 10 [cm] in length. Based on these premises, the elapsed time at each site of a NaV channel on the longitudinal line is calculated to be less than 10^−6^ [s] (= 10^−3^ [ms] = 1.0 [μs]).

(2) $$\begin{array}{r} Elapsed\;time\;at\;each\;NaV\;channel<10^{-6}\left[s\right] \end{array}$$

As we know, it takes more than 10^−1^ [ms] for an axon membrane to generate an action potential from the rise of the membrane potential (Platkiewicz and Brette, 2010). Based on these facts, the most advanced part of some kind of nerve conductive stimulant is already a longitudinal distance of more than 100 [channels] away from one specific NaV channel when a sufficient amount of ionic conductance and action potential are created around the channel. This means that the conduction in some form, which could trigger the opening of the NaV channels, passes much earlier than the generation of an action potential at each site of a NaV channel. This unexpected finding suggests that propagation of the nerve conduction happens without the generation of an action potential.

Based on these findings, in this report, we assume a new conductive model focusing on ionic migration based on Coulomb force inside the axon from a viewpoint different from the conventional equivalent circuit model.

### Generation of action potential

In the conventional cable theory, membrane potential at a specific coordinate and time on the membrane can be calculated by the Equation (1). However, as we described above, there is no actual metal leads spreading across the internal and external surfaces of the axonal membranes. The equivalent circuit model and cable theory are truly wonderful and splendid theories as a model to explain the phenomenon of action potential and nerve conduction. But in the actual sites of the internal surface of axons, there is no closed metal circuit in which the Ohm's law and Kirchhoff's law are realized. In the actual site, under the condition of steady state with no net charge transfer through the membrane, the membrane potential can be calculated by an equation using gradient of ionic concentrations along the axonal membrane as below, known as Goldman-Hodgkin-Katz (GHK) voltage equation (Goldman, 1943; Delamere and Duncan, 1977).

(3) $$\begin{array}{r} E_{s}=\left(\frac{R\;T}{F}\right)ln\frac{P_{K}{\left[K^{+}\right]}_{out}\;+\;P_{Na}{\left[Na^{+}\right]}_{out}\;+\;P_{Cl}{\left[Cl^{-}\right]}_{in}}{P_{K}{\left[K^{+}\right]}_{in}\;+\;P_{Na}{\left[Na^{+}\right]}_{in}\;+\;P_{Cl}{\left[Cl^{-}\right]}_{out}} \end{array}$$

(F: Faraday constant, R: the ideal gas constant, T: the temperature in kelvins, *P*_*ion*_: the permeability for each ion, [*ion*]_*in*_: the intracellular concentration of each ion, [*ion*]_*out*_: the extracellular concentration of each ion).

Though this equation does not stand when the steady state is broken, like the state of action potential, transfer of ions like sodium and potassium ions takes place in producing action potential. After the inflow of sodium ions at a specific NaV channel, actual change of ionic concentration around the next NaV channel is needed to make the membrane potential around the next NaV channel surpass the threshold of membrane potential to trigger the opening of the next NaV channel (Platkiewicz and Brette, 2010; Carter et al., 2012). This is one of the most basic and invariable principles in the generation of action potential, but could not be fully reflected in conventional theories based on Ohm's law and Kirchhoff's law.

As described before, in actual sites on an axonal membrane, the transmission of electric charges from one NaV channel to the next is realized not by the transmission of ion particles or free electrons all the way to the next NaV channel, but by the transmission of remote forces like Coulomb force from the inflowing mass of sodium ions to the ions around the next NaV channel. Without the change of ionic concentrations gradient across the membrane at the next NaV channel, at least only around the NaV channel, the generation of potential change around the next NaV channel or the opening of the next NaV channel would not be realized.

(4) $$\begin{array}{r} Generation\;of\;action\;potential\;\subset \\ \;Changes\;in\;ionic\;concentration\;gradient \end{array}$$

This relation is an important and fundamental principle, which was not fully considered in the conventional conduction models. In this paper, on the basis of this relation, we will focus on what the Coulomb force from a mass of inflowing sodium ions at one NaV channel will do to remote ion particles around the next NaV channel.

### Coulomb force and ionic migration around the next NaV channel

The initial velocity of ionic inflow from a NaV channel within very short period less than the first 1.0 [μs], an allowed limit of time period at one NaV channel as shown in the Equation (2), can be regarded as approximately constant, because the electrostatic repulsion from the formerly flown sodium ions to the later flowing sodium ions through a NaV channel can be disregarded within such a very short time period (Ohshima and Kondo, 1987; Chung and Kuyucak, 2002). Thus, we define the amount of electric charge of inflowing sodium ion at a specific NaV channel within a time period of 1.0 [ns] (= 10^−3^ [μs]) in the first 1.0 [μs] as a constant of +*Q*_1_ [C]. Then, the total amount of sodium ions flown through a NaV channel within the first *t*_1_ [ns] can be described as below.

(5) $$\begin{array}{r} \frac{dQ}{dt}\approx constant\;within\;the\;first\;1.0\;\left[\mu s\right]\\ ∴\;Electric\;charge\;flown\;through\;a\;NaV\;channel\;within\\ \;the\;first\;t_{1}\;\left[ns\right]\\ =\int_{0}^{t_{1}}Q_{1}\;dt=Q_{1}{\cdot t}_{1} \end{array}$$

Next, we define the elementary charges of monovalent cations and anions as a constant of ±*e* [C] (≈1.602 × 10^−19^ [C]). Because the ionic migration within fluid is quite slow and almost ignorable within a short period of nerve conduction, we can disregard the size of the inflowing mass of sodium ion from one NaV channel, compared to the distance from one NaV channel to the next. Then, the absolute values of the Coulomb forces from the mass of inflowing sodium ions (flown from 0 [ns] to *t* [ns]) to each of the cations and anions around the next NaV channel, which is *r* [m] away from the previous NaV channel, can be described as below.

(6) $$\begin{array}{rcl} \left|Repulsive\;Coulomb\;force\;for\;each\;cation\;\left[N\right]\right| & = & \frac{Q_{1}t\;e}{r^{2}} \end{array}$$

(7) $$\begin{array}{rcl} \left|Attractive\;Coulomb\;force\;for\;each\;anion\;\left[N\right]\right| & = & \frac{Q_{1}t\;e}{r^{2}} \end{array}$$

Here, there is an equation as below showing the ionic migration velocity and ionic flux (*J*) of a specific area in a specific time period within a fluid (Teorell, 1935). Ratio of ionic particles and water molecules is reflected in the value of the molar mobility (ω) and ionic concentration (*C*).

(8) $$\begin{array}{r} J\;\left[\frac{mol}{cm^{2}\cdot s}\right]=\omega CF \end{array}$$

(9) $$\begin{array}{r} Ionic\;migration\;velocity=\omega F=\frac{J}{C} \end{array}$$

*(*ω*: molar mobility, C: ionic concentration, F: driving force per gram-ion)*

Based on (6) and (7), Coulomb force to each ion (*F* [N]) at a specific distance from the mass of inflowing sodium ion particles is proportionate to *t* [ns]; thus, combined with (9), the following relationship is true.

(10) $$\begin{array}{r} Ionic\;migration\;velocity\;\propto \;F\;\left(Coulomb\;force\right)\;\propto \;t \end{array}$$

### Ionic movement along the axon membrane

Ion concentration in the intracellular fluid is roughly regarded as homogeneous throughout the axoplasm. Charge balance between cations and anions is preserved, even in the state of action potential, and the axoplasm in total is not strongly electrically-charged (Kurbel, 2008).

**Figure 2:**
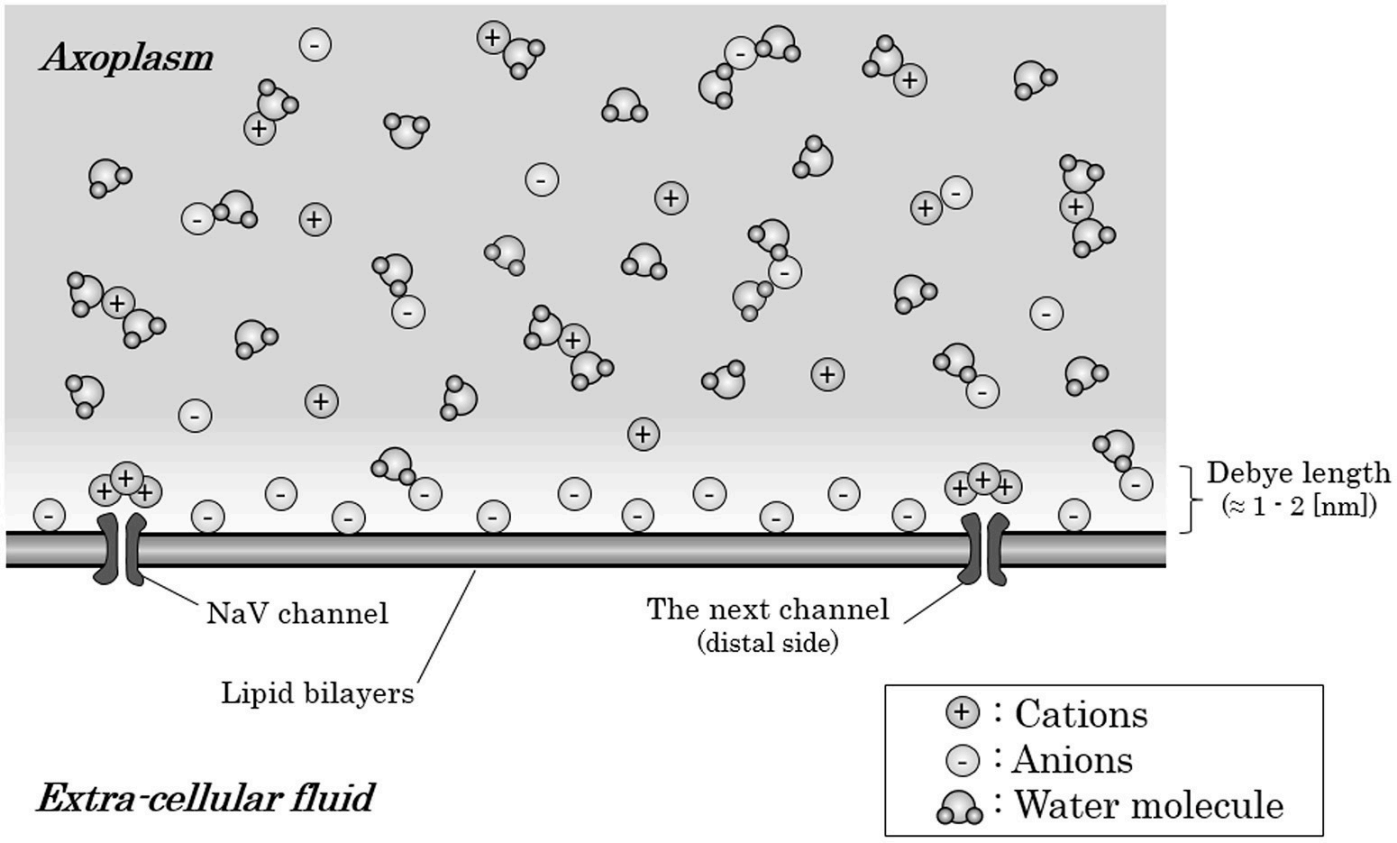
Ions and water molecules along the internal surface of an axon membrane. Within the Debye length (1–2 [nm]) from the internal surface of an axon membrane, anions are densely distributed; thus, this layer is negatively charged in total, only with the exception that cations crowded around the NaV channels which have negative charges on their surface. The axoplasm out of the Debye length from the surface are consisted of almost equal amount of anions and cations; thus, the axoplasm out of the Debye length is not electrically charged in total. Abbreviation: NaV, voltage-gated sodium ion channel.

But, strictly speaking, the cation-to-anion ratio within axoplasm is different based on the distance from the internal surface of axon membrane. The anion-densed layer on the internal surface of axon membrane is known to be restricted only within 1–2 [nm] from the internal surface, known as “Debye length” (Quinn et al., 1998; Bulai et al., 2012) (Figure 2). These anions within the Debye length from the internal surface of the membrane will be attracted by the Coulomb force from the inflowing sodium ion. The high density of water molecules, some of which hydrates the anions, and the ionic bands with cations would decelerate the ionic migration of the anions to some extent. However, the anions-densed horizontal layer within Debye length from membrane surface in total would be attracted by the Coulomb force and create a fluid flow toward the mass of inflowing sodium ions around the preceding NaV channel.

Here, there is an exception about the anion-crowded circumstance on the internal surface of axon membrane. Usually, cation channels have negative charges on their surfaces, which should produce an excess of cations near the internal surface of the axon membrane around NaV channels (Dani, 1986; Cukierman et al., 1988; Miedema, 2002).

**Figure 3:**
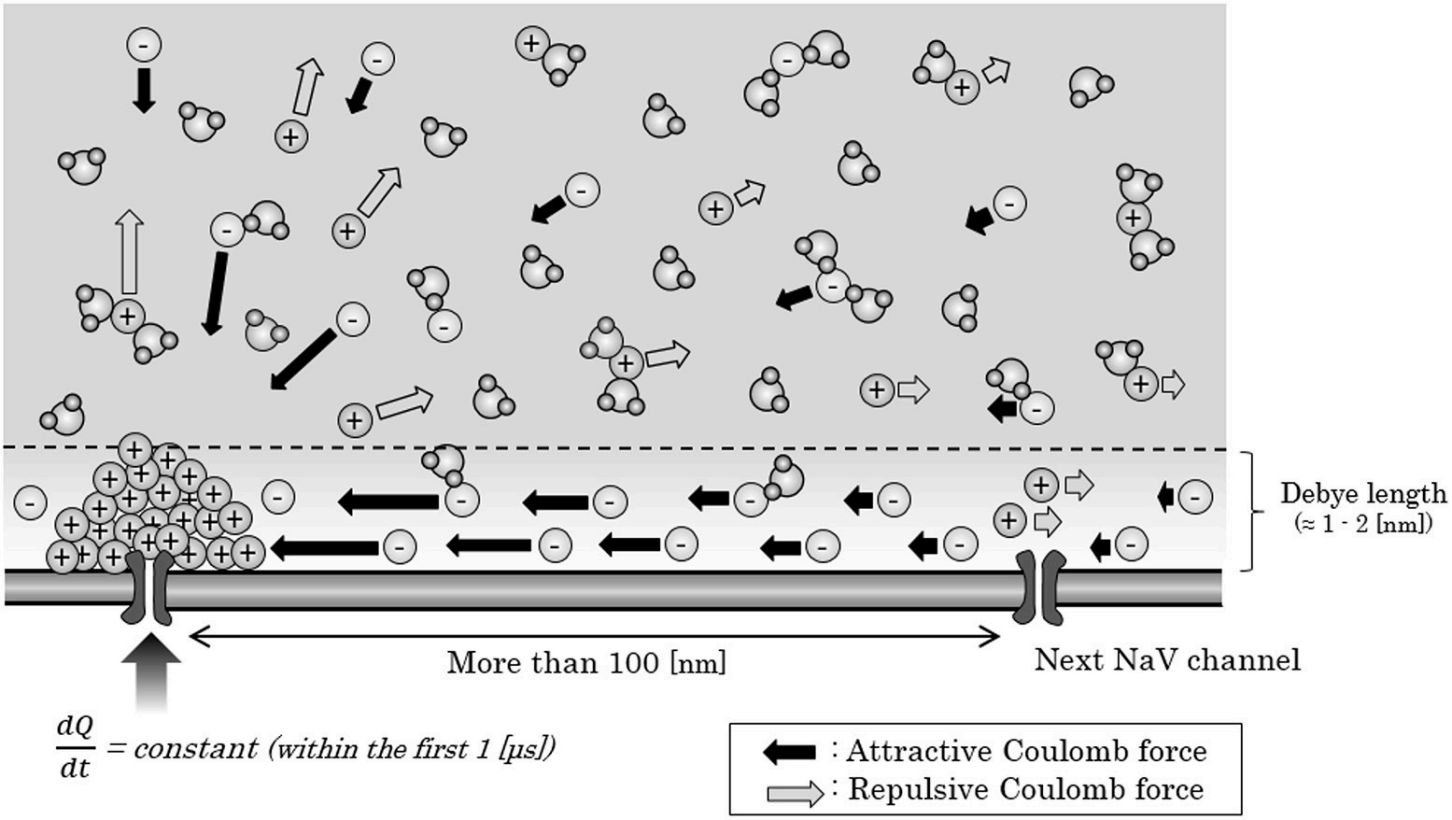
Ionic migration by Coulomb force from the inflowing sodium ions. When a mass of sodium ions are created around a NaV channel in the process of the generation of an action potential, ions in the axoplasm would receive an attractive or repulsive Coulomb force from the mass. Within the Debye length, electric charge at each site would not be changed, except for the minute space around the next NaV, where the cations crowded around the negatively-charged surface of NaV. Abbreviations: dQ, small change in the amount of inflowing electric charges; dt, very short time period; NaV, voltage-gated sodium ion channel.

**Figure 4:**
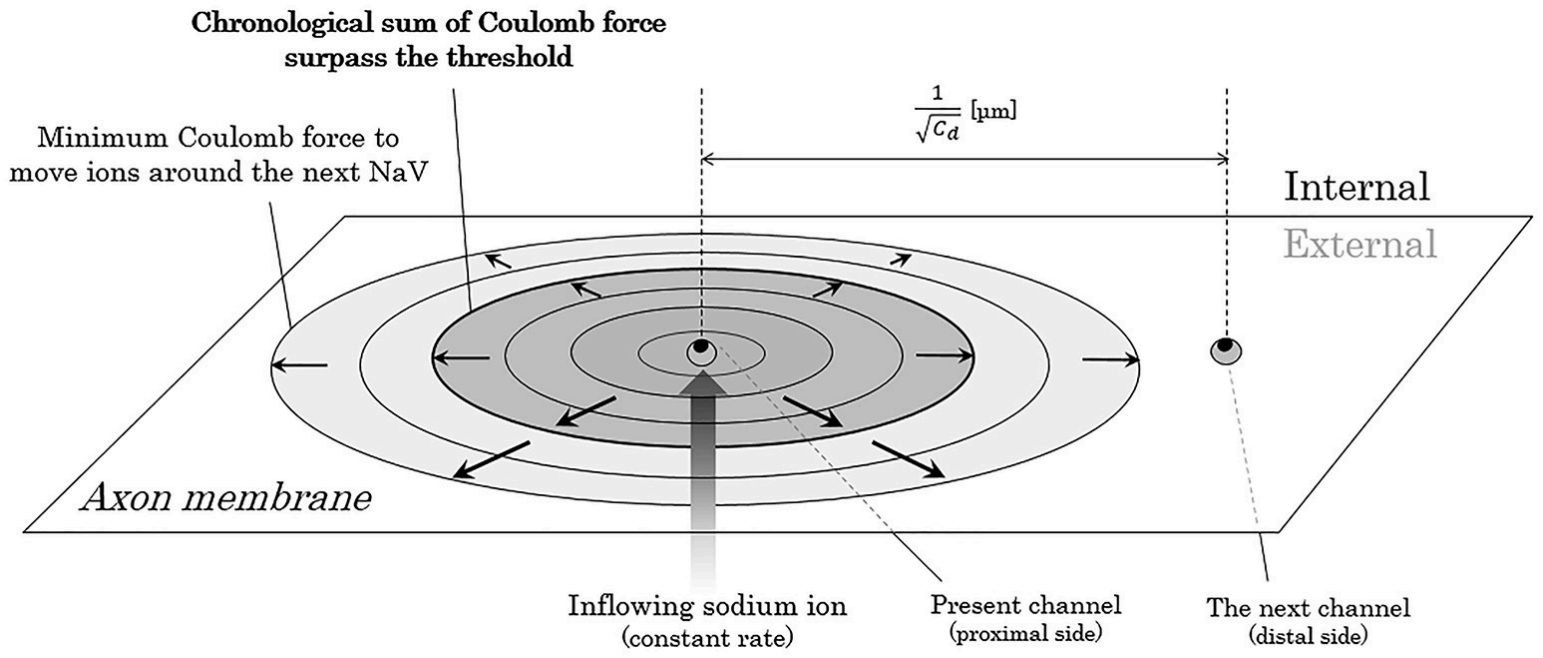
Propagation of the threshold line of Coulomb force to trigger opening of the next NaV. The closer the electric charges are, the stronger the Coulomb force between them. Thus, the ion particles close to the mass of inflowing sodium ions receive stronger Coulomb force than the ion particles far away from the mass. The threshold forefront line of Coulomb force to trigger opening of the next NaV expands as the inflowing sodium ions increases by time. Abbreviation: *C*_*d*_, channel density of voltage-gated sodium ion channel on the membrane.

Now, we consider about the situation when a mass of sodium ion particles flowing through a NaV channel from extracellular fluid to axoplasm (Figure 3). As shown in the figure, the bulk of inflowing sodium ion affect charged ions in axoplasm; cations will receive repulsive forces and anions will receive attractive forces. Because of this inverse direction of Coulomb force between anions and cations, cation-to-anion ratio within the Debye length in the limited space around the next NaV channel will be dramatically changed by the Coulomb force from inflowing sodium ion. If the accumulated sum of the Coulomb force surpass the minimum threshold and the ionic milieu around the next NaV channel has been sufficiently changed, it causes conformational change on the structure of next NaV channel and triggers its opening. Propagation of the forefront line of such minimum threshold in Coulomb force can be illustrated as shown in Figure 4. As described later, threshold Coulomb force to trigger opening of the next NaV channel differs based on the channel density, in other words, based on the distance of two-adjacent NaV channels.

### Size of axons

The diameters of axons in unmyelinated nerves are generally known to be smaller than those in myelinated nerves (Ikeda and Oka, 2012). Usually, the diameter of an axon in unmyelinated nerves is 0.2–1.2 [μm] (Ritchie, 1982). In this study, the diameter of the axon is described as “*D*” [μm], which is between 0.2 and 1.2. By using this variable, the cross-sectional periphery and area of an axon can be described as below.

(11) $$\begin{array}{r} Cross-sectional\;circumference\;=\pi \;D\;\left[\mu m\right] \end{array}$$

(12) $$Cross-sectional\;area\;=\frac{\pi \;D^{2}}{4}\;[\mu m^{2}]$$

Based on (11) and (12), if the axon is cut with the length of *L* [μm], the cell membrane area and the intracellular volume of the cut axonal section can be described as below.

(13) $$Area\;of\;cell\;membrane\;[\mu m^{2}]\;=\pi \;D\;L\;[\mu m^{2}]$$

(14) $$Intracellular\;volume\;[\mu m^{3}]=\;\frac{\pi \;D^{2}\;L}{4}\;[\mu m^{3}]$$

### Ratio of intracellular ions and inflowing sodium ion

The membrane capacitance is known to be around the below-mentioned value (Gentet et al., 2000; Golowasch et al., 2009).

$$Membrane\;capacitance\;\approx 1.0[\frac{\mu F}{cm^{2}}]\;=\;10^{-14}[\frac{C}{V^{*}\mu m^{2}}])$$

Thus, to produce an electric potential of 100 [mV] (= 0.1 [V]) on the axon membrane with an area of π*DL* [μm^2^] (axon with *L* [μm] in length, *D* [μm] in diameter), a total amount of π*DL* × 10^−15^ [C] of electric charge must be transferred from the extracellular to intracellular fluid. The electric charge of one particle of monovalent cation is 1.602 × 10^−19^ [C]; thus, to produce an action potential, a minimum of the following number of sodium ion particles must be transferred.

(15) $$\begin{array}{r} Transferred\;ion\;particles\;to\;produce\;action\;potential\\ \;\approx \frac{\pi \;D\;L}{1.602\;\times \;10^{-4}} \end{array}$$

Here, the usual concentrations of intracellular sodium and potassium ions are around 15 [mEq/l] and 140 [mEq/l], respectively. The numbers of intracellular cation particles (sodium and potassium) in the same length of an axon before the inflow of sodium ions can be estimated with the following equations, using the Avogadro constant of 6.02 × 10^23^ [mol^−1^]:

(16) $$\begin{array}{r} Intracellular\;cations\;\left[particles\right]\;\approx \;\left(140+15\right)\;\times \;10^{-3}\times \\ \left(6.02\times 10^{23}\right)\times \;\frac{\pi \;D^{2}\;L\;10^{-15}}{4}\;=\left(2.\text{33 }\times 10^{7}\right)\cdot \pi \;D^{2}L \end{array}$$

Thus, the ratio of inflowing sodium ion particles to the intracellular cation particles before the inflow is as follows:

(17) $$\begin{array}{r} Sodium\;ion\;influx:\;intracellular\;cations\;\approx \text{ 1 }:\;\left(3.\text{73 }\times \;10^{3}\times \;D\right) \end{array}$$

This ratio shows that the number of intracellular cation particles originally present before the sodium influx is far greater than the number of sodium ion particles producing the action potential, more than 1,000 times higher than sodium influx; this suggests that sodium influx through the NaV channel would not significantly change the intracellular concentration of cations. Based on this result, in this study, we ignore the effect of Coulomb force from the inflowing sodium ions on different sides of the axon membrane, and only focus on each pair of two longitudinally-adjacent NaV channels.

### Density and distance of sodium ion channels on an axonal membrane

The density of NaV channels in unmyelinated nerves is known to be 5-50 [channels/μm^2^], and mainly differs depending on the distance from the axon hillock, the root of an axon (Hu and Jonas, 2014). For convenience, in this study, channel density in one axon is regarded as being constant. Here, we define the channel-density of NaV channels on an axon membrane as *C*_*d*_.

$$\begin{array}{l} Density\;of\;NaV\;channels\;on\;an\;axon\;membrane\;=C_{d} \end{array}$$

**Figure 5:**
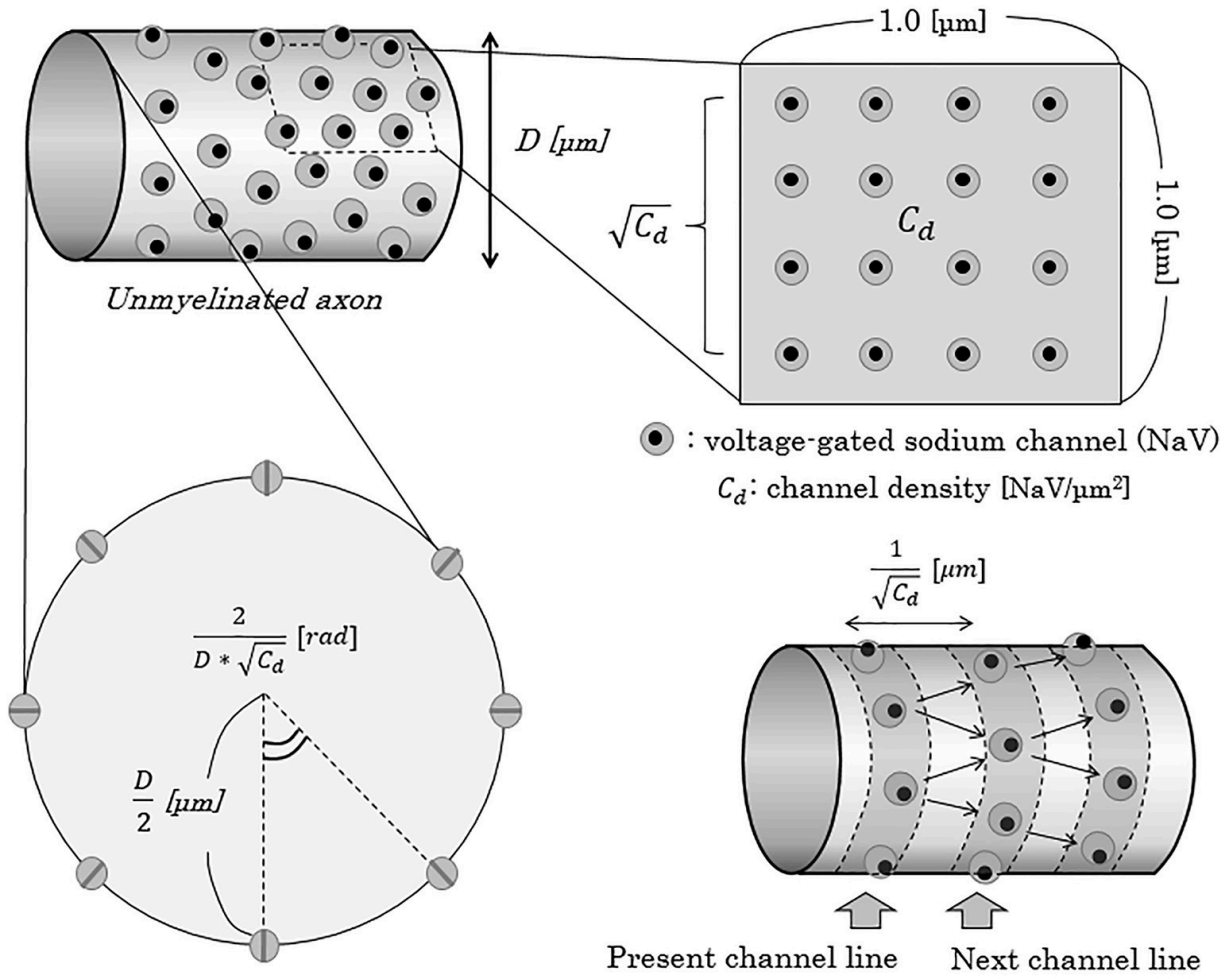
Schema of the concept of NaV channel density and distribution on the axonal membrane. When the density of NaV channels is *C*_*d*_ [channels/μm^2^], the distance of the adjacent NaV channel line from one channel line can be described as 1/$\sqrt{C_{d}}$ [μm]. In this report, for convenience, we adapted a model of “NaV channel line”, parallel to the cross-section of axons, and regarded an axon as a pile of such channel lines. *C*_*d*_, NaV channel density [channels/μm^2^]; D, diameter of axon [μm]; NaV, voltage-gated sodium channel; rad, radian.

To mathematically describe the distance from one NaV channel to the next NaV channel on distal (peripheral) side, we apply a model in which the NaV channels evenly align circularly on one cross section of an axon, expressed as “NaV channel line” with a ring formation. Then, distance from one channel line to the next adjacent channel line can be described as below (Figure 5).

(18) $$\begin{array}{r} Distance\;between\;two\;adjacent\;NaV\;channel\;lines\;\left[\mu m\right]=\\ \;\frac{1}{\sqrt{C_{d}}} \end{array}$$

The theoretical relationship between the axonal diameter (*D*) and NaV channel density (*C*_*d*_) will be discussed later.

On a cross-section of one NaV channel line of an axon with *D* [μm] (0.2 ≤ *D* ≤ 1.2) in diameter, the numbers of NaV channels and the central angle comprised by the two adjacent NaV channels can be described as below.

(19) $$\begin{array}{r} Number\;of\;NaV\;channel\;on\;one\;channel\;line\;\left[channels/section\right]\\ =D\;\pi \;\sqrt{C_{d}} \end{array}$$

(20) $$\begin{array}{r} Central\;angle\;between\;two\;adjacent\;NaV\;channels\;\left[rad\right]\\ =\frac{2}{D\;\sqrt{C_{d}}} \end{array}$$

### Conduction velocity

The nerve conduction in unmyelinated nerves can be theoretically described as the integration of continuous transmission of an action potential, realized by changes in ionic concentrations around NaV channels, from one NaV channel to the next. Thus, the average time period to take for nerve conduction along a specific distance can be calculated by the average time lapse from opening of one NaV channel to opening of the next adjacent NaV channel (*t*_*adjacant*_) multiplied by the number of channel lines existing along the specific distance.

(21) $$\begin{array}{r} <Time\;to\;take\;for\;nerve\;conduction\;along\;a\;specific\;length>\\ =t_{adjacent}\cdot <numbers\;of\;NaV\;channel\;lines\;along\;a\;specific\;length> \end{array}$$

In this equation, angle brackets (< >) means the average on time of the content.

### Theoretical relationship between the axonal diameter and channel density

Until now, there has been no study showing the relationship between the axonal diameter (*D*) and channel density (*C*_*d*_). In this report, we will theoretically estimate the relationship based on the following conditions.

1. Because there is no organelle inside axons, NaV protein is translated in the neuronal body and then transported by kinesin protein on the rail of microtubules in axon.
2. As previously known, the numbers of microtubule in axons are proportionate to the axonal cross-sectional area [μ*m*^2^]. (Hoffman et al., 1985; Iturriaga, 1985).
3. Sufficient amounts of NaV channels are expressed in the neuronal body in normal condition, so that the rate of gene expression does not affect or control the rate of NaV-channel transportation to the efferent side.

In a state of equilibrium of the channel density, the rate of NaV channel importation and NaV channel breakdown are equal in a specific area of the axon membrane [μm^2^]. Based on the third condition, the total number of imported NaV channels inside the axon can be proportionate to the number of microtubules inside the axon. Based on these premises, we will consider the theoretical relationship between an axonal diameter and a channel density.

### Other assumptions

In the model of this report, which mainly focuses on the relationship between the axonal diameter and conduction velocity, factors other than the axonal diameter (*D*) and channel density (*C*_*d*_), like temperature, density of sodium ion channels on cell membrane, resistivity of the fiber interior, membrane capacitance per unit area, or the unit area resistance of the membrane in its excited state) were regarded as constant, regardless of the axonal diameter (Matsumoto and Tasaki, 1977).

## Results

### Distance of two longitudinally-adjacent NaV channels

**Figure 6:**
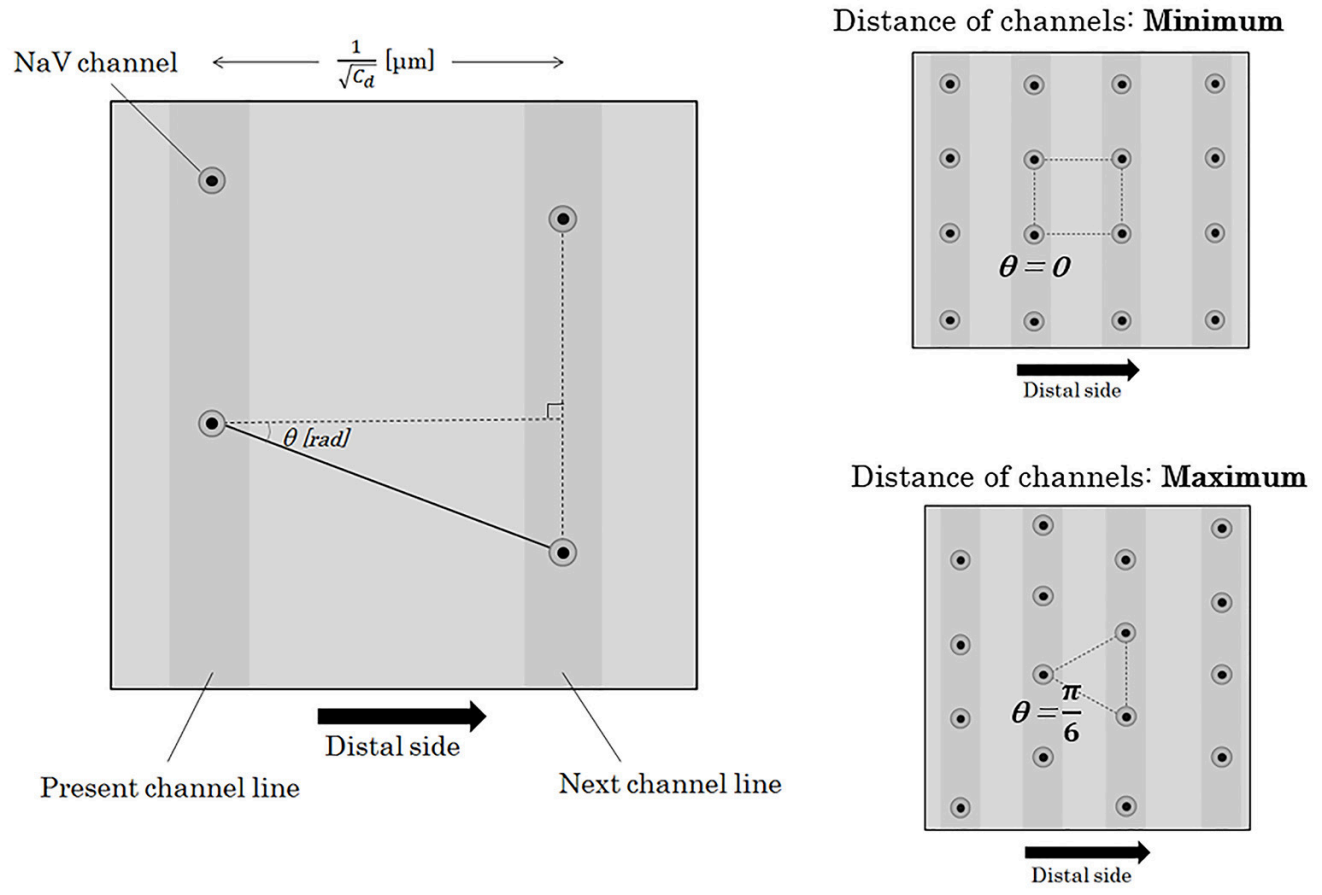
Distance between two adjacent NaV channels. If we define the angle between the longitudinal line parallel to the running of an axon and the line connecting two longitudinally nearest NaV channels as θ, the distance between the two adjacent channels on the different channel lines can be described as ${\left(\sqrt{C_{d}}\cdot cos\theta \right)}^{-1}$. The angle of θ takes values between –π/6 and +π/6 [rad]. Abbreviations: *C*_*d*_, channel density; NaV, voltage-gated sodium ion channel.

Here, we will consider about the distance between a NaV channel and the next on the nex*t c*hannel line. On the surface of an axon membrane, the angle (θ [rad]) between an axonal longitudinal line and the line connecting a NaV channel with the next on the next channel line can be distributed evenly between −π/6 [rad] and +π/6 [rad] (Figure 6). The probability density function of θ [rad] (Φ*(*θ*)*) shows a homogeneous distribution with a constant probability as below.

(22) $$\begin{array}{r} \Phi \left(\theta \right)=\frac{3}{\pi }\left(-\frac{\pi }{6}\;\leq \;\theta <\frac{\pi }{6}\right) \end{array}$$

Here, we divide θ into 2 sections at θ = *0* [rad] and further divide each of the 2 sections into *n*-sections with a regular interval (*n*: natural number). The probability of the *n*-components (*n*-possibilities) of the next NaV channel location is even. Distance from a NaV channel to the *k*-th component (*k* = *0,1,2,3,…,n*) among the *n*-possibilities of the next NaV channel can be described as below.

(23) $$\begin{array}{r} Distance\;between\;a\;NaV\;channel\;to\;the\;next\\ \;=\frac{1}{\sqrt{C_{d}}}\cdot \frac{1}{\cos\left(\frac{k}{n}\cdot \frac{\pi }{6}\right)} \end{array}$$

If we see the total length of an axon, the distribution of θ is totally random with enough numbers of integration. The heterogeneous distribution of θ at each pair of two adjacent NaV channel lines can be homogenized in the total length of the axon. The average of the distance between a NaV channel and the next on the next channel line can be calculated as below. The angle brackets (< >) means the average on time of the content.

(24) $$\begin{array}{rc} <Distance\;from\;a\;NaV\;channel\;to\;the\;next>\\ = & \underset{n\to \infty }{\lim}\left(\frac{1}{n}\right)\sum_{k=1}^{n}\left[\frac{1}{\sqrt{C_{d}}}\cdot \frac{1}{\cos\left(\frac{k}{n}\cdot \frac{\pi }{6}\right)}\right]\\ = & \int_{0}^{1}\left[\frac{1}{\sqrt{C_{d}}}\cdot \frac{1}{\cos\left(\frac{\pi }{6}\cdot x\right)}\right]dx\\ = & \frac{\pi }{6}\cdot \frac{1}{\sqrt{C_{d}}}\int_{0}^{\frac{\pi }{6}}\left(\frac{1}{\cos\varphi }\right)d\phi \\ = & \frac{\pi }{12}\cdot \frac{1}{\sqrt{C_{d}}}{\left[ln\frac{1+sin\varphi }{1-sin\varphi }\right]}_{0}^{\frac{\pi }{6}}\\ =\frac{\pi \cdot ln3}{12\;\sqrt{C_{d}}}\;\propto \;\frac{1}{\sqrt{C_{d}}} \end{array}$$

### Time of the coulomb force to trigger opening of the next NaV

**Figure 7:**
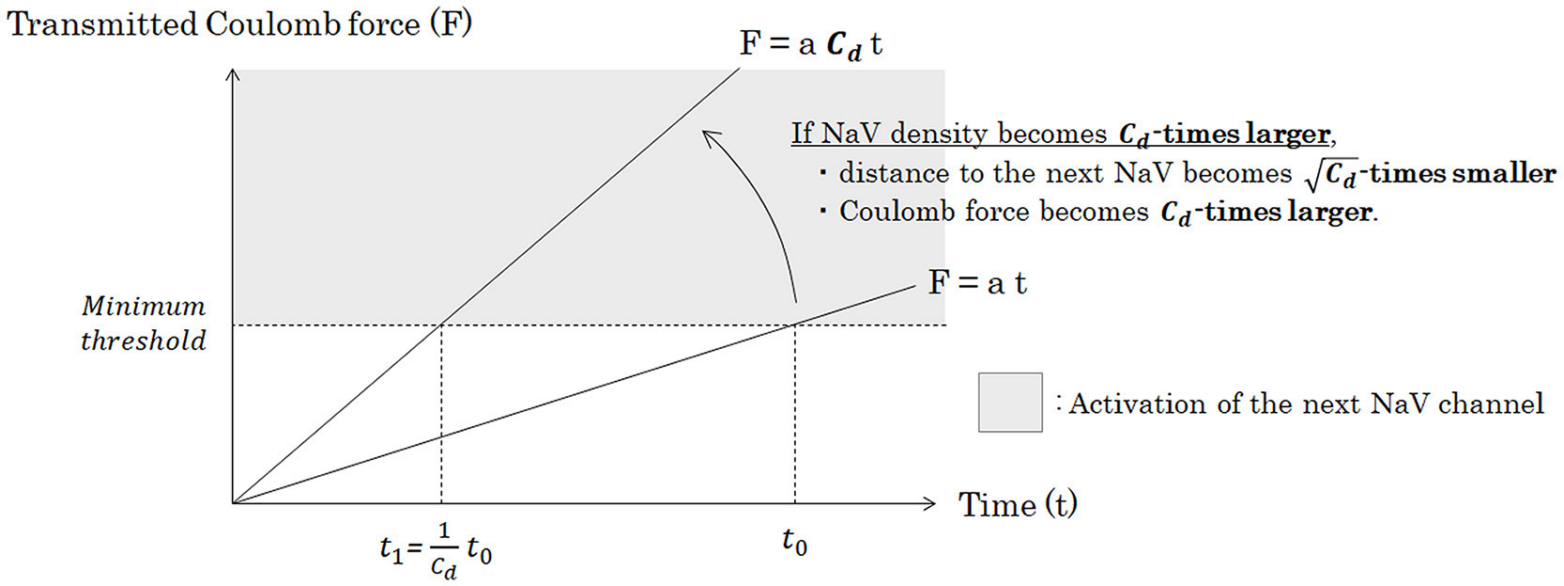
Relationship between the density of NaV channels (*C*_*d*_) and the time of the Coulomb force to open the next NaV channel. Under a specific pattern of NaV distribution and a condition of constant inflowing rate of sodium ion through a NaV, Coulomb force (F) from inflowing sodium ion mass to ion particles around the next NaV is proportionate to time. Accumulation of the transmitted Coulomb force within a specific time is proportionate to the strength of Coulomb force, if the inflowing rate of sodium ion is constant. Thus, if the channel density becomes *C*_*d*_-times larger, time from the opening of a NaV channel to trigger the opening of the next NaV channel will be *C*_*d*_-times shortened. Abbreviations: *C*_*d*_, NaV channel density [channels/μm^2^]; NaV, voltage-gated sodium channel.

Under the same pattern of distribution of NaV channels on the membrane, as a matter of course, conduction velocity is regulated by the longitudinal amount of the NaV channels to be transmitted and the average time from one NaV channel to the next. As can be seen in Figure 7, when the channel density becomes *C*_*d*_-times larger, the transmitted Coulomb force between adjacent NaV channels becomes *C*_*d*_-times larger, and the time to surpass the minimum threshold to trigger the opening of next NaV channel will be *C*_*d*_-times shortened.

(25) $$\begin{array}{r} t_{1}\;=\;\frac{1}{C_{d}}\;t_{0} \end{array}$$

In other words, we can conclude that average time lapse from opening of one NaV channel to opening of the next NaV channel (*t*_*adjacent*_) is inversely proportionate to *C*_*d*_.

(26) $$\begin{array}{r} t_{adjacent}\;\propto \;\frac{1}{C_{d}} \end{array}$$

### Conduction velocity

Based on the equation of (21), we will consider about the relationship between the channel density (*C*_*d*_) and the conduction velocity. The numbers of NaV channel lines along the specific length is proportionate to the square root of the channel density.

(27) $$\begin{array}{r} <numbers\;of\;NaV\;channel\;lines\;along\;a\;specific\;length>\\ \;\propto \sqrt{C_{d}} \end{array}$$

Here, the time to take for nerve conduction along a specific length can be calculated by the time from one NaV to the next multiplied by the longitudinal number of NaV, as shown in the Equation (21). Thus, based on (21), (26), and (27), the following relation is true.

(28) $$\begin{array}{r} <Time\;to\;take\;for\;nerve\;conduction\;along\;a\;specific\;length>\\ \;\propto \;\frac{1}{\sqrt{C_{d}}} \end{array}$$

Now, the conduction velocity of a nerve can be described as the reciprocal of the average time to take for the nerve conduction along a specific length. Thus, the following relation about the conduction velocity of unmyelinated nerves is true.

(29) $$\begin{array}{rcl} Conduction\;velocity\; & = & \frac{1}{<time\;to\;take\;along\;a\;specific\;length>}\\ \propto \sqrt{C_{d}} \end{array}$$

### Relationship between an axonal diameter and channel density

Based on the three conditions proposed in the corresponding section in the methods section, we will consider about the mathematical relationship between an axonal diameter (*D*) and a channel density (*C*_*d*_). In a steady state of the normal condition, the rate of breakdown of NaV channels and the rate of importation of NaV channels are equilibrated on a specific area of the axon membrane.

(30) $$\begin{array}{rc} Rate\;of\;NaV\;breakdown\;in\;a\;specific\;ranges\;of\;time\;and\;area\\ = & \;Rate\;of\;NaV\;importation\;in\;a\;specific\;ranges\;of\;time\;and\;area \end{array}$$

The rate of NaV channel breakdown in a specific range of time and area can be proportionate to the channel density (*C*_*d*_) in the specific area.

(31) $$\begin{array}{r} Rate\;of\;NaV\;breakdown\;in\;a\;specific\;range\;of\;time\\ and\;area\;\propto \;C_{d} \end{array}$$

The rate of NaV channel importation in a specific range of time and area can be roughly estimated by the total number of transported NaV channels inside the axon divided by the specific area of the axonal membrane [μm^2^].

(32) $$\begin{array}{r} Rate\;of\;NaV\;importation\;in\;a\;specific\;range\;of\;time\;and\;area\\ \propto \frac{Total\;amount\;of\;transported\;NaV\;channels\;inside\;axon}{Area\;of\;axonal\;membrane\;\left[\mu m^{2}\right]}\\ \propto \;\frac{\pi \cdot {\left(\frac{D}{2}\right)}^{2}}{2\pi \cdot \frac{D}{2}}\propto D \end{array}$$

Based on (30), (31), and (32), we can theoretically establish the following relationship.

(33) $$\begin{array}{r} C_{d}\;\propto \;D \end{array}$$

Combined with (29), conduction velocity of unmyelinated nerves can be theoretically described as below.

(34) $$\begin{array}{r} Conduction\;velocity\;\propto \;\sqrt{C_{d}}\;\propto \;\sqrt{D} \end{array}$$

## Discussion

In this report, we saw a possible new model of nerve conduction in unmyelinated nerves from viewpoints of NaV channels and Coulomb forces between ion particles, apart from the concept of the conventional electrical circuit model. The conventional concept of equivalent circuit model and cable theory were absolutely wonderful models, both of which well reproduced the electric phenomenon happening along the axonal membrane (Hodgkin and Huxley, 1952). However, the previous model did not fully accommodate the principles that the axoplasm is consisted of randomly migrating ion particles and water molecules, without closed circuit of metal leads to transmit electrical potential, and that action potential cannot be generated without preceding change of ionic concentration gradient across the axon membrane. Also, as we saw in the methods section about the allowed time lapse in one NaV channel, the velocity of nerve conduction much exceeds the time scale of the generation of action potential at each NaV channel. The allowed time lapse at each NaV channel is the scale of nano-seconds, but the generation of action potential at each NaV channel takes place in the time scale of micro-seconds to milli-seconds (Carter and Bean, 2011). Based on this fact, the generation of action potential in nerve conduction would be just a secondary phenomenon, not an essential driving force of the nerve conduction. The essential driving force of the nerve conduction in unmyelinated nerves would be a remote propagation of the changes in intracellular ionic concentrations which is principally produced by the Coulomb force from the inflowing sodium ions through NaV channels.

In this report, conduction velocity in an unmyelinated nerve was theoretically shown to be proportionate to the square root of the axonal diameter based on a new model mainly focusing on simple geometric arguments. This conclusion matches not only the previous experimental data but also the previous theoretical reports, all of which described that conduction velocity in unmyelinated nerves would be proportionate to the square root of the axonal diameter (Hodgkin, 1954; Bucher and Goaillard, 2011). Compared to the conventional theory by Hodgkin and Huxley to explain the relationship between conduction velocity and axonal diameter, our new model have some strengths. The conventional theory was fundamentally based on equivalent circuit model, which adopts Ohm's law and Kirchhoff's law to the actual site of wet biological environment. Besides, the conventional theory adopted a presumptive condition that action potential would propagate with a constant conduction velocity, which would be not always guaranteed in the actual site. In our new model, we only presume Coulomb force from one NaV channel to the next and only use basic arithmetic operations without any kinds of differential equations.

We have to discuss about the Coulomb force on the ion particles around a NaV channel from the NaV channels on previous channel lines. Strictly speaking, the ion particles around a NaV channel receive the Coulomb force not only from the inflowing sodium ions from the NaV channel on the adjacent previous channel line but also from NaV channels on more forward channel lines. However, in this report, we only focused on the Coulomb force from the NaV channel on the adjacent previous channel line. This was because the sum of the Coulomb force from all the passed NaV channels on the previous channel lines could be proportionate to the Coulomb force from the NaV channel on the adjacent previous channel line as shown below. By focusing on a longitudinal section parallel to the axon running, based on the Equations (6) and (7), an increment of the total Coulomb force within 1.0 [ns] for an ion particle on a specific channel line from the ion particles from all the passed NaV channels (Δ*Q*_*SUM*_) can be described as below.

$$\begin{array}{lll} \left|\Delta Q_{SUM}\right| & \propto & \frac{Q_{1}}{{\left(1\cdot \sqrt{C_{d}}\right)}^{2}}+\frac{Q_{1}}{{\left(2\cdot \sqrt{C_{d}}\right)}^{2}}+\frac{Q_{1}}{{\left(3\cdot \sqrt{C_{d}}\right)}^{2}}\\ +\;\frac{Q_{1}}{{\left(4^{\cdot }\sqrt{C_{d}}\right)}^{2}}+\cdot \cdot \cdot \\ = & \left(\frac{Q_{1}}{C_{d}}\right)\cdot \sum_{n\;=\;1}^{\infty }\frac{1}{n^{2}}\\ = & \left(\frac{Q_{1}}{C_{d}}\right)\cdot \frac{\pi^{2}}{6}\\ \propto \;Q_{1}\;\left(\text{if }C_{d}\text{ is constant}\right) \end{array}$$

Based on this result, when we think about the Coulomb force on ion particles around a specific NaV channel, we can only focus on the inflowing sodium ions from the adjacent previous NaV channel and we can conclude that the Equations (24) and (26) still stand even when we strictly consider about the Coulomb force from all the passed NaV channels in addition to the adjacent previous NaV channel.

Lastly, to be emphasized, this report does not deny the conventional theories of nerve conductions like equivalent circuit model and cable theory. Those conventional theories are well-established models and can wonderfully explain the phenomenon taking place on axon membranes. This new model, proposed in this paper, is just an additional and supportive concept for those conventional theories from a different viewpoint of ion channels and the Coulomb force between ion particles. By combining the conventional viewpoint of electric circuit and this new viewpoint of ionic migration and Coulomb force, we would be able to achieve more accurate and easier understanding of the phenomenon in nerve conduction.

There are some limitations for this study. First, we did not pathologically prove the theoretical relation of “*C*_*d*_∝*D.”* This relation is desired to be pathologically reconfirmed in the future. Another limitation is that we do not consider the effects from factors other than axonal diameter and NaV channel density in this study. Certainly, there are some variables that have been theoretically suggested to affect nerve conduction like temperature, resting membrane potential, membrane resistance, and membrane capacitance (Debanne et al., 2011). Theoretical explanation to connect these variables with the efficiency of nerve conduction will be the subjects in the future. And the last limitation is that our new model does not fully cover the physiological phenomena in myelinated nerves with myelin sheathes, in which the conduction velocity is known to be roughly proportionate to the axonal diameter, not the square root of it. We need a totally different theoretical model to explain the nerve conduction in myelinated nerves.

In conclusion, by applying new conductive model, which is mainly focused on the transfer of membrane potential by the Coulomb force from the inflowing sodium ions at a NaV channel to the ions around the next NaV channel, we could theoretically estimate and reproduce the phenomenon happening in unmyelinated nerve conduction. By combining this new viewpoint from geometric distribution of ion channels and Coulomb force with the conventional electric circuit model, we would be further able to theoretically explain the actual phenomenon in unmyelinated nerves.

## Author contributions

The author confirms being the sole contributor of this work and approved it for publication.

### Conflict of interest statement

The author declares that the research was conducted in the absence of any commercial or financial relationships that could be construed as a potential conflict of interest.
